# A Computational Analysis of Bone Formation in the Cranial Vault in the Mouse

**DOI:** 10.3389/fbioe.2015.00024

**Published:** 2015-03-19

**Authors:** Chanyoung Lee, Joan T. Richtsmeier, Reuben H. Kraft

**Affiliations:** ^1^The Penn State Computational Biomechanics Group, Department of Mechanical and Nuclear Engineering, Pennsylvania State University, University Park, PA, USA; ^2^Department of Anthropology, Pennsylvania State University, University Park, PA, USA

**Keywords:** computational morphogenesis, finite volume method, skull growth, developmental biology, skull sutures

## Abstract

Bones of the cranial vault are formed by the differentiation of mesenchymal cells into osteoblasts on a surface that surrounds the brain, eventually forming mineralized bone. Signaling pathways causative for cell differentiation include the actions of extracellular proteins driven by information from genes. We assume that the interaction of cells and extracellular molecules, which are associated with cell differentiation, can be modeled using Turing’s reaction–diffusion model, a mathematical model for pattern formation controlled by two interacting molecules (activator and inhibitor). In this study, we hypothesize that regions of high concentration of an activator develop into primary centers of ossification, the earliest sites of cranial vault bone. In addition to the Turing model, we use another diffusion equation to model a morphogen (potentially the same as the morphogen associated with formation of ossification centers) associated with bone growth. These mathematical models were solved using the finite volume method. The computational domain and model parameters are determined using a large collection of experimental data showing skull bone formation in mouse at different embryonic days in mice carrying disease causing mutations and their unaffected littermates. The results show that the relative locations of the five ossification centers that form in our model occur at the same position as those identified in experimental data. As bone grows from these ossification centers, sutures form between the bones.

## 1 Introduction

**Figure 1:**
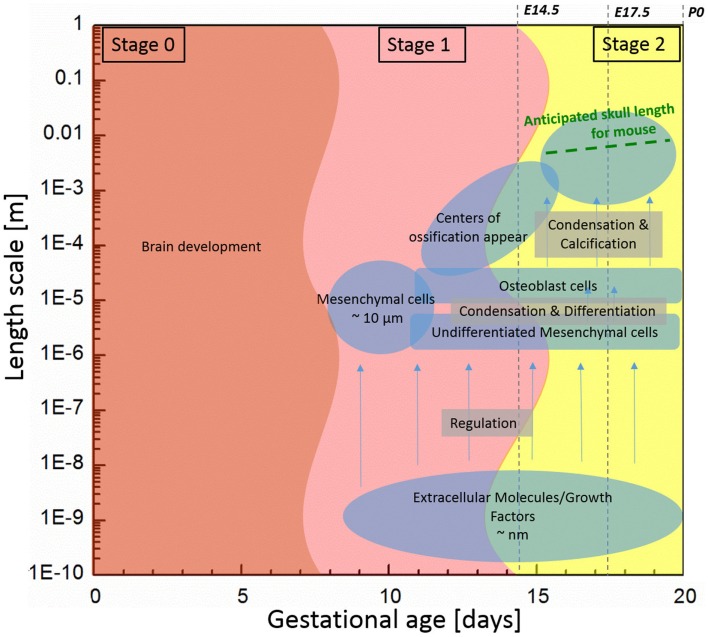
**Components associated with generalized mouse cranial vault bone development ranging from molecules to tissue according to time and length scale**. Data are generated from observations of C57Bl6 mice.

Bones of the mammalian cranial vault are formed by the process of intramembranous ossification where condensations of multipotent mesenchymal cells differentiate directly into functioning osteoblasts to form bone (Tubbs et al., 2012; Percival and Richtsmeier, 2013). Figure 1 shows the estimated length and time scales associated with various processes of skull development in the mouse. The study of the formation of cranial vault bones is critical because it may help uncover fundamental mechanisms associated with birth defects such as craniosynostosis (involving premature closure of cranial vault sutures and skull dysmorphology) as well as answer basic questions in developmental biology and evolution.

Initially, undifferentiated mesenchymal cells migrate to future sites of bone formation situated on the brain and its meningeal layers (pia, arachnoid, dura mater). Over time, the proliferation and differentiation are regulated by growth factor signaling pathways and their downstream transcription factors in order for these cells to become committed to an array of different fates. In some cells, the intracellular signaling pathways cause differentiation of mesenchymal cells into osteoblasts, the cells that build bone (Marie et al., 2002; Gordeladze et al., 2010; Tubbs et al., 2012). Differentiation of condensed groups of osteoblasts results in the formation of ossification centers that form in tissue membranes surrounding the brain. Next, osteoblasts begin to secrete a bone matrix, osteoid, which is then mineralized, eventually forming a bone of the cranial vault. For the mouse, the process of cell migration begins around embryonic day 9 (E9) and skull bone growth continues postnatally, involving length scales ranging from the nanometer to millimeter as schematically shown in Figure 1.

**Figure 2:**
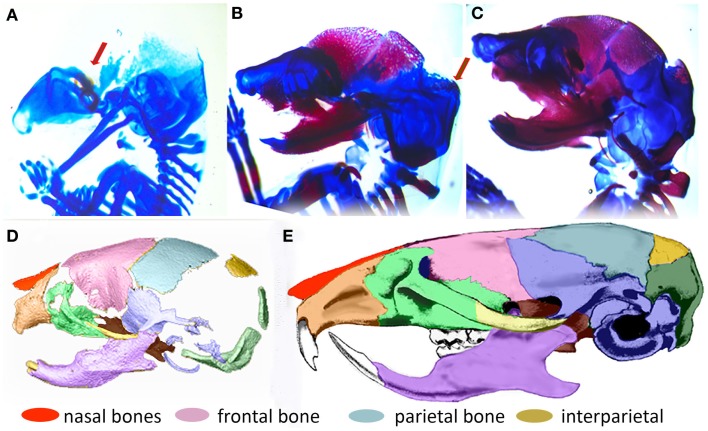
**Formation of the mouse skull**. **(A–C)** Histological images of developing mouse embryos showing formation of bone (magenta stained by alizarin red) and cartilage (stained by alcian blue) of the embryonic skull. **(A)** lateral view of embryonic mouse head at embryonic day 14.5 (E14.5) showing site of initial ossification of the frontal bone of the cranial vault (red arrow) and skull cartilage; **(B)** at E15.5, the forming frontal and parietal bones are clearly visible and the ossification of the interparietal bone is beginning (arrow); **(C)** By E17.5, the interparietal is well formed and other bones of the skull are clearly visible. **(D)** 3D reconstruction of micro computed tomography image of the mouse skull at birth (P0) colored to indicate the placement and level of maturity of all skull bones. **(E)** Adult mouse skull colored to show relative position of skull bones.

In order to understand the fundamental mechanisms of skull growth, both experimental and computational methods have been employed. Many studies have experimentally studied the roles of various proteins in cranial bone formation (Holleville et al., 2003; Wan and Cao, 2005) and growth of cranial bones (Martínez-Abadías et al., 2013; Motch Perrine et al., 2014; Percival and Richtsmeier, 2013) and compared the craniofacial bone growth patterns of normal mice and those carrying mutations that in humans cause disease. Figure 2 shows that cranial vault bone elements (frontal, parietal, and interparietal bones) appear from embryonic day 14.5 (E14.5) and continue to grow through postnatal day 0 (P0) and beyond. It also shows that the frontal bone forms first and the interparietal bone forms later, forming sutures between individual bones while they grow. Experimental studies are extremely valuable but can be costly and only so many variations can be explored. Therefore, it is valuable to also examine the possibility of using computational methods to understand fundamental mechanisms of morphogenesis.

Several computational studies have been conducted to model the process of skull bone formation. A mathematical model for reaction–diffusion controlled by two interacting chemical molecules, proposed by Turing (1952), has been employed in the study of biological pattern formation and development of biological systems. Kondo and Shirota (2009) analyzed the mechanism of skin pattern formation of animals using the Turing model and (Marcon and Sharpe, 2012) adopted the model to explain various biological development processes. Garzón-Alvarado et al. (2013) used the model to establish a computational framework for investigating bone formation in human cranial vault. The model, commonly referred to as the reaction–diffusion model, shows that through the regulatory loop of interacting molecules the concentration of the molecules forms an inhomogeneous special pattern in space. In this study, we adopt an approach similar to that of Garzón-Alvarado et al. (2013), to study growth of the skull in a mouse model of human disease and then propose an extension of the framework.

As depicted on Figure 1, we subdivide the process into two stages: (1) initiation (differentiation) of primary centers of ossification; and (2) bone growth. In the first stage, we focus on differentiation of osteoblast lineage cells (OLCs), which leads to the initial primary centers of ossification of the flat bones of the cranial vault. We assume that the interaction of extracellular molecules, which are associated with the differentiation process of OLCs along an osteogenic path, can be modeled using the reaction–diffusion model. Reaction–diffusion models can be further subdivided into activator–inhibitor and activator–substrate models according to how molecules interact with each other (Gierer and Meinhardt, 1972). The primary difference between the two models is the way one molecule inhibits the other molecule. In the activator–inhibitor model, one molecule (activator) enhances the other molecule (inhibitor) but the enhancement of inhibitor inhibits the action of activator so that the molecules are in phase. On the other hand, in the activator–substrate model, one molecule (activator) consumes the other molecule (substrate) to be enhanced and eventually is restricted by depletion of the other molecule (substrate) so that the molecules are not in phase. More details about these models can be seen in a work of Koch and Meinhardt (1994). Since many details remain to be discovered in order to fully understand the key molecular players in proliferation and differentiation of OLCs as they form intramembranous bones of the cranial vault, it is currently not known whether the activator–inhibitor or the activator–substrate approach more accurately models intramembranous bone formation. Since the activator–inhibitor model more closely models what has been observed experimentally and because the regulatory relation in the model is simpler, unlike Garzón-Alvarado et al. (2013), we employ the activator–inhibitor model.

In the second stage, we deal with the rapid proliferation of bone cells from the primary condensations and their outward migration (stage 1) to form bones of the cranial vault, particularly the frontal and parietal bones. Work from other laboratories collectively suggest a pattern of presumptive bone cells expanding outward from mesenchymal condensations, predominantly towards the apex of the head (Iseki et al., 1997; Rice et al., 2000; Ting et al., 2009). Under normal regulatory conditions, these expanding condensations represent the primary region of osteoblast differentiation, the source of differentiating cells, and locus of osteogenesis and bone growth. They also define the earliest shapes of developing bones. We modeled the action of the morphogen stimulating bone growth from these osteoblasts.

In this paper, we present a combined experimental and computational study of cranial bone growth. In Section 2, we present the computational framework for determining the location of primary ossification centers and simulating growth of individual bones of the cranial vault. Our computational predictions are guided and compared to a large archive of experimental data. Micro computed tomographic (*μCT*) images of mouse heads of various embryonic ages were used for making a computational domain and testing and tuning the mathematical model by comparison between experimental and computational results. Section 3 gives results of the simulation and discussions about them are made in Section 4. Finally, in Section 5, we conclude and summarize our findings and provide suggestions for future work.

## 2 Materials and Methods

### 2.1 Mathematical model

#### 2.1.1 Regulation of osteoblast differentiation

Osteoblasts differentiate from mesenchymal progenitors, going through distinct developmental stages, which are regulated by various developmental signals. Although the role of key developmental signals is well defined, most of this knowledge comes from studies of endochondral development of long bones and even there, little is known about how the signals execute the osteoblast-specific differentiation program (Long, 2011).

This study is based on the hypothesis that differentiation of mesenchymal cells takes place by chemical reactions triggered by the interaction of extracellular molecules (both local and systemic regulatory signals), with cell surface ligands, although it is important to point out that the specific proteins in the mouse system are not clearly determined. Thus, we quickly arrive at one challenge of this approach: determining quantitative values of the parameters that represent specific proteins. We acknowledge that parameters are the cause of great speculation throughout this study and maintain that developing this model as part of a collaborative project that seeks to precisely define the role of the critical molecular participants will improve our model over time. Conversely, it is clearly a challenge to obtain and measure these parameters experimentally, and we propose that computational studies presented here will help elucidate possible bounds of parameters and guide the design of future experiments to determine their values with increased certainty. To mitigate the speculative nature of parameterization, we conduct a stability analysis that helps to bound some values and parametric studies to estimate the effects of some of the parameters.

**Figure 3:**
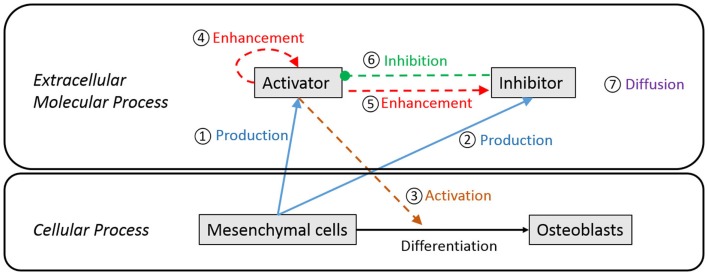
**Schematic diagram of extracellular and cellular process associated with differentiation of mesenchymal cells to osteoblast cells**. Undifferentiated mesenchymal cells surrounding the brain express diffusible extracellular molecules, which play a key role in cell differentiation (① and ②). One of the molecules (activator) activates signaling pathways to initiate cell differentiation of mesenchymal cells into osteoblasts (③). In an extracellular process, the activator simultaneously enhances itself (④) and the other key molecule (inhibitor) (⑤), while the inhibitor inhibits the activator (⑥). These two proteins eventually establish a regulatory loop and diffuse in space with different speed (⑦) to form an inhomogeneous spatial pattern of concentration.

**Figure 4:**
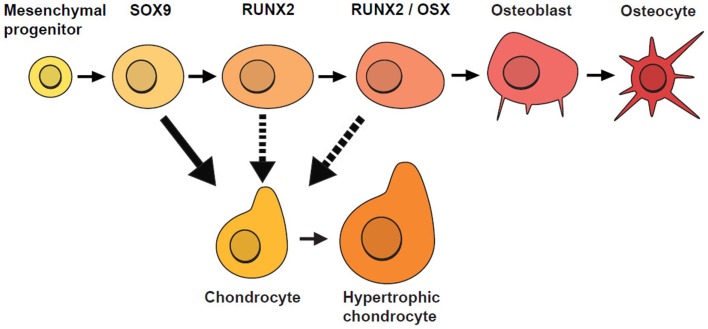
**Stages of osteoblast lineage cell (OLC) differentiation**. Mesenchymal progenitors give rise to osteoblasts and chondrocytes and are usually marked by SOX9. If SOX9+ cells do not differentiate into chondrocytes, they progress along an osteogenic path and as they mature, they are marked progressively by the expression of RUNX2, followed by OSX. Additional evidence suggests that SOX9+ cells can switch fates under certain conditions (indicated by dotted lines) [adapted from Long (2011)].

**Figure 5:**
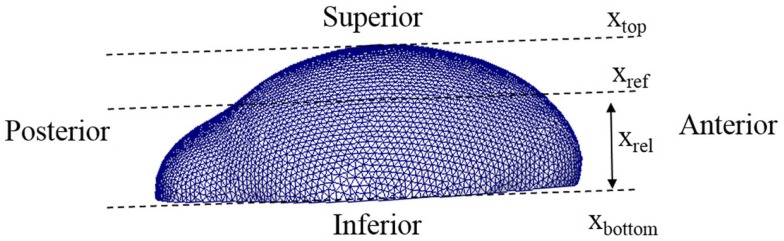
**Lateral view of a 3D domain**. The relative distance from inferior surface of the domain (*x_rel_*) and reference position (*x_ref_*) are indicated. Our model limits differentiation to those cells below the reference position.

**Figure 6:**
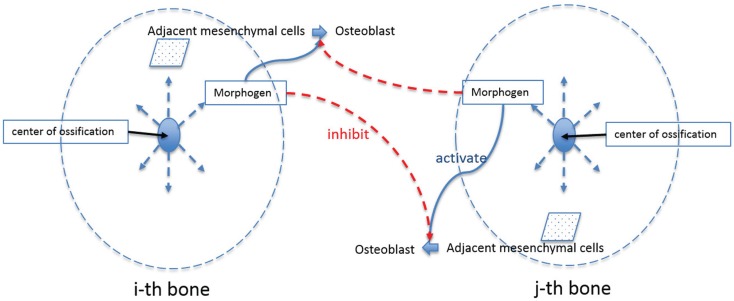
**Schematic diagram of bone growth from primary ossification centers**. Osteoblasts in the ossification centers begin to secret a morphogen, which diffuses into the neighboring space and allows adjacent mesenchymal cells to differentiate into osteoblasts. Suture formation between bone elements is modeled by assuming that differentiation of mesenchymal cells adjacent to bone is restricted by the morphogen secreted from the adjacent bone elements.

**Figure 7:**
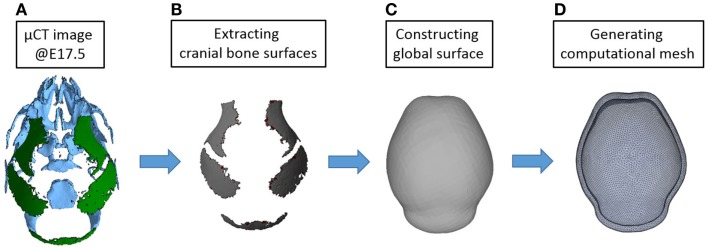
**Procedure for constructing a computational domain**. **(A)** An isosurface of the cranial vault bones from a three-dimensional reconstruction of micro CT images acquired from a mouse at E17.5 was used for making the domain and generating a mesh. Rostral end (nose) is toward the top while the caudal end of the skull is at bottom. **(B)** Only parts of cranial vault are considered. **(C)** Surface surrounding the cranial vault is made. **(D)** Shell-shaped domain around the surface and mesh are generated.

Figure 3 is a schematic diagram showing the process of differentiation of mesenchymal cells to osteoblasts and the regulatory relationship between extracellular molecules, which influence cell differentiation. In our model, mesenchymal cells surrounding the brain express diffusible extracellular molecules, which play a key role in cell differentiation and the molecules are assumed to follow behaviors that can be described using the activator–inhibitor model (Figure 3, ① and ②). The activator initiates chemical reactions that activate signaling pathways to initiate differentiation of mesenchymal cells into osteoblasts (Figure 3, ③). In an extracellular process, the activator simultaneously enhances itself (Figure 3, ④) and the inhibitor (Figure 3, ⑤), while the inhibitor inhibits the activator (Figure 3, ⑥). These two proteins eventually establish a regulatory loop and the diffusion of products of the inhibitor and activator (Figure 3, ⑦) form an inhomogeneous spatial pattern of concentration that results in molecules condensing at specific locations in a domain. The features of an activator–inhibitor system can be mathematically modeled using a reaction–diffusion model, which is given by:

(1a) $$\frac{\partial C_{a}}{\mathrm{\partial t}}=\sigma_{a}-\mu_{a}C_{a}+\rho_{a}\frac{C_{a}^{2}}{C_{h}}+D_{a}\nabla^{2}C_{a}$$

(1b) $$\underset{①}{\underset{︸}{\frac{\partial C_{h}}{\partial t}}}=\underset{②}{\underset{︸}{\sigma_{h}}}-\underset{③}{\underset{︸}{\mu_{h}\;C_{h}}}+\underset{④}{\underset{︸}{\rho_{h}\;C_{a}^{2}}}+\underset{⑤}{\underset{︸}{D_{h}\;\nabla^{2}\;C_{h}}}$$

where, *C_a_* and *C_h_* represent the concentration of activator and inhibitor, respectively. *σ_a_* and *σ_h_* are the constants quantifying the production of activator and inhibitor from mesenchymal cells. The parameters *μ_a_* and *μ_h_* quantify degradation or depletion of the proteins. The parameters *ρ_a_* and *ρ_h_* are constants associated with the non-linear interaction between activator and inhibitor. The interaction term shows that the activator enhances itself and the inhibitor [*C_a_*^2^ in numerator in equations (1a) and (1b)] but is constrained by the inhibitor [*C_h_* in denominator in equation (1a)]. *D_a_* and *D_h_* represent the diffusion rate of each molecule and ▽^2^ is the Laplace operator $\left(i\mathrm{.e}.,\sum_{i=1}^{3}\frac{\partial^{2}}{\partial {x_{i}}^{2}}\right)$ describing the spatial diffusion of molecules. So equations (1a) and (1b) show that the time rate of change of concentration of each molecule [equations (1a) and (1b)-①] is determined by its production from mesenchymal cells [equations (1a) and (1b)-②], degradation [equations (1a) and (1b)-③], interaction between the two molecules [equations (1a) and (1b)-④] and diffusion into space [equations (1a) and (1b)-⑤].

In this model, parameters should satisfy a certain constraint in order to make an inhomogeneous spatial pattern from a very small perturbation on a homogeneous initial condition. If diffusion of a molecule is fast relative to the reaction between activator and inhibitor, a small perturbation cannot be amplified but the molecules will reach another homogeneous condition. In the future, actual values of parameters might be defined by way of laboratory experiments but experiments that quantify these types of parameters are currently limited. Therefore, parameters should be estimated with careful consideration of the biologically reasonable range. Additionally, Koch and Meinhardt (1994) suggested constraints that the parameters should satisfy for pattern formation using a linear stability analysis. Homogeneous, steady state initial concentration of each molecule can be achieved mathematically by setting time rate of change and spatial diffusion terms in equations (1a) and (1b) be to zero:

(2) $$C_{a0}=\frac{\mu_{h}\rho_{a}}{\mu_{a}\rho_{h}}+\frac{\sigma_{a}}{\mu_{a}},\;C_{h0}=\frac{\rho_{h}}{\mu_{h}}C_{a0}^{2}$$

where *C_a0_* and *C_h0_* signify the concentration of activator and inhibitor, respectively, at steady state. By adding a small perturbation to the homogeneous steady condition, the concentration of two molecules can be represented as equations (3a) and (3b) where,

(3a) $$C_{a}=C_{a0}+\delta C_{a},\;C_{h}=C_{h0}+\delta C_{h}$$

(3b) $${\delta C}_{a}=\delta C_{a0}e^{\omega t}\cos\left(2\pi \mathrm{kx}\right),\;\delta C_{h}=\delta C_{h0}e^{\omega t}\cos\left(2\pi \mathrm{kx}\right)$$

In equation (3b) perturbation *δC_a_* and *δC_h_* are assumed to change in time and space. The value *ω* can be a complex number and the imaginary part of it represents a frequency at which the perturbation changes in time. The change in space is characterized by wave number *k*, which is associated with *ω*. When the real part of the frequency *ω* is positive, the perturbation increases with time so that concentrations of molecules can form an inhomogeneous spatial pattern. By substituting equations (2), (3a), and (3b) into equations (1a) and (1b) and conducting a linear stability analysis, a condition, which parameters should satisfy for pattern formation (i.e., for making the real part of *ω* to be positive) can be obtained as below:

(4) $$\frac{2\mu_{h}\rho_{a}}{\mu_{h}\rho_{a}+\sigma_{a}\rho_{h}}-1\leq \frac{\mu_{h}}{\mu_{a}}<\frac{D_{h}}{D_{a}}{\left(\sqrt{\frac{2\mu_{h}\rho_{a}}{\mu_{h}\rho_{a}+\sigma_{a}\rho_{h}}}-1\right)}^{2}$$

Details can be found in Koch and Meinhardt (1994). In this study, we used a set of parameters, which meet the condition of equation (4) as listed in Table 1. Using values in Table 1 with equation (4) yields: 0.667 < 2.0 < 8.468. The effects of various model parameters on the results can be seen in Supplementary Material.

**Table 1:** **Model parameters in reaction–diffusion model of activator and inhibitor**.

Parameters		Value	Units
Activator production	*σ_a_*	1.0 × 10^−5^	ng/(mm^3^ × s)
Inhibitor production	*σ_h_*	1.0 × 10^−5^	ng/(mm^3^ × s)
Activator depletion	*μ_a_*	5.0 × 10^−5^	/s
Inhibitor depletion	*μ_h_*	1.0 × 10^−4^	/s
Activator coupling	*ρ_a_*	5.0 × 10^−5^	/s
Inhibitor coupling	*ρ_h_*	1.0 × 10^−4^	mm^3^/(ng × s)
Activator diffusion	*D_a_*	2.5 × 10^−6^	mm^2^/s
Inhibitor diffusion	*D_h_*	2.5 × 10^−4^	mm^2^/s

Similar to Garzón-Alvarado et al. (2013), our model is based on the hypothesis that differentiation of mesenchymal cells into osteoblasts is triggered by an activator. Here, we acknowledge that there are several biological stages prior to the final differentiation of OLCs into osteoblasts (Figure 4), so our model focuses on cellular mechanisms occurring after the concentration of OLCs has formed and the cells have started along an osteoblast differentiation trajectory. These cells are more likely to form osteoblasts where concentration of activator is high. In addition to concentration of activator, spatial positions of cells are also found to affect the rate of cell differentiation. Tubbs et al. (2012) shows that cells on the inferior surface (that will form bones of the skull base) differentiate faster than cells on the superior surface (cranial vault bones). With these impacting factors on cell differentiation the rate of generation of osteoblast can be modeled by the equations (5a) and (5b).

(5a) $$\underset{①}{\underset{︸}{\frac{\partial C_{O}}{\partial t}}}=\eta \underset{②}{\underset{︸}{\frac{C_{a}{\;}^{n}}{C_{a}{\;}^{n}+C_{aT}{\;}^{n}}}}\underset{③}{\underset{︸}{\frac{T_{a}{\;}^{n}}{T_{a}{\;}^{n}+t^{n}}}}\underset{④}{\underset{︸}{f\left(x_{rel}\right)}}$$

(5b) $$f\left(x_{\mathrm{rel}}\right)=H\left(x_{\mathrm{ref}}-x_{\mathrm{rel}}\right)=\{\begin{array}{cc} 1 & x_{rel}\leq x_{ref}\\ 0 & \mathrm{otherwise} \end{array}$$

In the equations, *C_o_* represents the concentration of osteoblast and *C_a_* indicates the concentration of activator as before. *C_aT_* represents the threshold concentration of activator that allows mesenchymal cells to differentiate, which means only cells in the region where the concentration of activator exceeds the threshold value can differentiate into osteoblasts. *T_a_* represents the time limit of action of the activator, in other words after this time is reached the action of the activator decreases. The value *η* is a constant quantifying the amount of osteoblast generated by action of activator. The value *n* is a constant characterizing how sharply the rate of cell differentiation increases after the concentration of activator reaches the threshold value. The function *f*(*x_rel_*) is a term about spatial effect on cell differentiation and limits differentiation to those cells below the reference position (*x_ref_*). This function can be represented by Heaviside function as shown in equation (5b). The relative distance from inferior surface of the domain (*x_rel_*) and reference position (*x_ref_*) are shown in Figure 5. So equations (5a) and (5b) show that the rate of cell differentiation [equation (5a)-①] is determined by concentration of activator [equation (5a)-②], elapsed time in the differentiation process [equation (5a)-③] and spatial position [equation (5a)-④]. Numerical values of the model parameters about cell differentiation used in this study are arranged in Table 2.

**Table 2:** **Model parameters in cell differentiation model**.

Parameters		Value	Units
Differentiation constant	*η*	1.0 × 10^−5^	ng/(mm^3^ × s)
Activator threshold	*C_aT_*	6.0	ng/mm^3^
Activator time limit	*T_a_*	4.0 × 10^2^	hr
Control constant	*n*	8.0	
Reference position	*x_ref_*	1.85	mm
Maximum position	*x_top_*	2.49	mm

#### 2.1.2 Bone growth

After the formation of primary ossification centers, essentially condensations of osteoblasts differentiated from mesenchymal cells, bone grows from these centers by a combination of the production of osteoid and its mineralization by osteoblasts and continued differentiation of rapidly proliferating mesenchymal cells that migrate outwards from the condensation (Iseki et al., 1997; Rice et al., 2000; Yoshida et al., 2008; Ting et al., 2009). The process can be shown schematically in Figure 6. Osteoblasts in the ossification centers begin to secret various morphogens. Some of these morphogens (e.g., Dlx5) are produced from osteoblasts and diffuse into the neighboring space allowing adjacent mesenchymal cells to differentiate into osteoblasts. Experimental data reveal in mice that five primary ossification centers, one each for the bones of the cranial vault (right and left frontal, right and left parietal and interparietal bones) that appear first on the more rostral surface of the embryonic mouse brain as shown in Figure 2. The squamous portion of the occipital bone develops later. In normal conditions, the bones of the skull vault are separated by sutures, fibrous joints that accommodate the expanding brain and allow the skull to undergo reshaping during birth (Ting et al., 2009). Although the mechanisms of suture formation are only partly known, most research favors molecular mechanisms that balance the proliferation and differentiation of osteogenic cells in the developing sutures (Lee et al., 1999; Yousfi et al., 2001, 2002; Chen et al., 2003; Bialek et al., 2004) and/or that establish tissue boundaries (Merrill et al., 2006; Ting et al., 2009) in suture formation and premature closure of sutures. Similar to Garzón-Alvarado et al. (2013) in this study, suture formation between bone elements is modeled by assuming that differentiation of mesenchymal cells adjacent to bone is restricted by the morphogen secreted from the adjacent bone elements, although we realize that the signaling could come from, or be transmitted through dura mater surrounding the developing brain (Opperman, 2000; Richtsmeier and Flaherty, 2013). The differentiation process in the bone growth stage is modeled by following equation in this study.

(6) $$\underset{①}{\underset{︸}{\frac{\partial C_{o-i}}{\partial t}}}=\underset{②}{\underset{︸}{\lambda \frac{C_{oS-i}{\;}^{n}}{C_{o-i}{\;}^{n}+C_{oS-i}{\;}^{n}}}}\underset{③}{\underset{︸}{\frac{C_{m-i}{\;}^{n}}{C_{m-i}{\;}^{n}+C_{mT-i}{\;}^{n}}}}\;\times \;\underset{④}{\underset{︸}{\prod_{j=1;j\neg i}^{5}\frac{C_{ml-j}{\;}^{n}}{C_{m-j}{\;}^{n}+C_{ml-j}{\;}^{n}}}}$$

In this equation, *C_o_* indicates the concentration of osteoblast and subscription *i* indicates any quantities of *i*-th bone where *i* can represent the number of different regions of skull bone (in our case, 5 regions: right and left frontal, right and left parietal and interparietal bone). *C_os_* is saturation concentration of osteoblast that sets the upper limit of generation of osteoblast. *C_m_* represents the concentration of the morphogen, which is secreted from osteoblasts and allows differentiation of adjacent mesenchymal cells. *C_mT_* is the threshold value of the morphogen that triggers differentiation of mesenchymal cells. *C_ml_* in equation (6) represents the limitation concentration of the morphogen, in which cell differentiation of *i*-th bone is restricted when it senses morphogen above this value secreted from *j*-th bone (*i* ≠* j*). *λ* is a constant quantifying the amount of osteoblast differentiated by action of the morphogen. *n* is a constant characterizing how sharply the rate of cell differentiation increases after the concentration of the morphogen reaches the threshold value, or decreases after concentration of osteoblast and morphogen from other regions of bone elements reach a saturation and limitation value, respectively. So equation (6) says that the rate of cell differentiation in one bony element [equation (6)-①] is determined by saturation control by itself [equation (6)-②], action of the morphogen allowing cell differentiation [equation (6)-③], and restriction by the morphogen from other bony elements [equation (6)-④].

In our model, the production and diffusion of the morphogen associated with bone growth are modeled by another diffusion equation:

(7) $$\underset{①}{\underset{︸}{\frac{\partial C_{m-i}}{\partial t}}}=\alpha \underset{②}{\underset{︸}{C_{o-i}}}\underset{③}{\underset{︸}{\frac{C_{mS-i}{\;}^{n}}{C_{m-i}{\;}^{n}+C_{mS-i}{\;}^{n}}}}+\underset{④}{\underset{︸}{D_{m-i}\nabla^{2}C_{m-i}}}$$

In this equation, *C_m_* represents the concentration of the morphogen. *C_o_* indicates the concentration of osteoblasts from which the morphogen is expressed. *C_mS_* is the saturation concentration of the morphogen, in which, production of the morphogen is restricted after this value. *D_m_* represents diffusion rate of the morphogen and ▽^2^ is the Laplace operator describing spatial diffusion of the morphogen. *α* is a constant quantifying the amount of the morphogen expressed by osteoblasts. *n* is a constant characterizing how sharply the expression of the morphogen decreases after the concentration of the morphogen reaches the saturation value. So equation (6) shows that the time rate of change of the morphogen concentration [equation (7)-①] is affected by concentration of already differentiated osteoblasts [equation (7)-②], which secrete the morphogen, restriction when it reaches saturation [equation (7)-③] and diffusion into space occupied by undifferentiated mesenchymal cells [equation (7)-④]. The model parameters used with equations (6) and (7) are listed in Table 3.

**Table 3:** **Model parameters in bone growth model**.

Parameters		Value	Units
Morphogen constant	α	5.0 × 10^−5^	/s
Morphogen saturation	*C_mS_*	2.0	ng/mm^3^
Morphogen diffusion	*D_m_*	1.2 × 10^−7^	mm^2^/s
Growth constant	*λ*	1.0 × 10^−1^	ng/(mm^3^ × s)
Osteoblast saturation	*C_oS_*	1.0	ng/mm^3^
Morphogen threshold	*C_mT_*	2.0	ng/mm^3^
Morphogen limitation	*C_ml_*	1.0 × 10^−3^	ng/mm^3^

### 2.2 Numerical implementation

The finite volume method using the open source code OpenFOAM 2.2.2 was applied to solve the reaction–diffusion model of activator and inhibitor, cell differentiation model, and bone growth model [equations (1a), (1b), (5a), (5b), (6), and (7)].

#### 2.2.1 Computational domain

In our previous study, the reaction–diffusion model of activator and inhibitor (BMP2 and Noggin in the paper) was solved in a one-dimensional and two-dimensional domain (Lee et al., 2014). In this study, we used three-dimensional reconstructions of micro CT images of mouse heads at embryonic day 17.5 (E17.5) to conduct a computational simulation on a more realistic domain. At E17.5, some parts of mouse skull are already formed as shown in Figure 7. We considered only bones of the cranial vault and simplified them to make a surface on which reaction–diffusion of extracellular molecules takes place. Then we made a shell-shaped domain around the surface and generated a mesh using commercial software ANSYS ICEM. The length of the vault is approximately 7 mm and the thickness of the shell is about 0.3 mm. In total, 84,200 elements are used for the calculation. In future applications, we will move these simulations to surfaces of earlier embryonic ages before bone is formed to more realistically model the domain prior to the initiation of cranial vault bone formation.

#### 2.2.2 Initial condition

**Figure 8:**
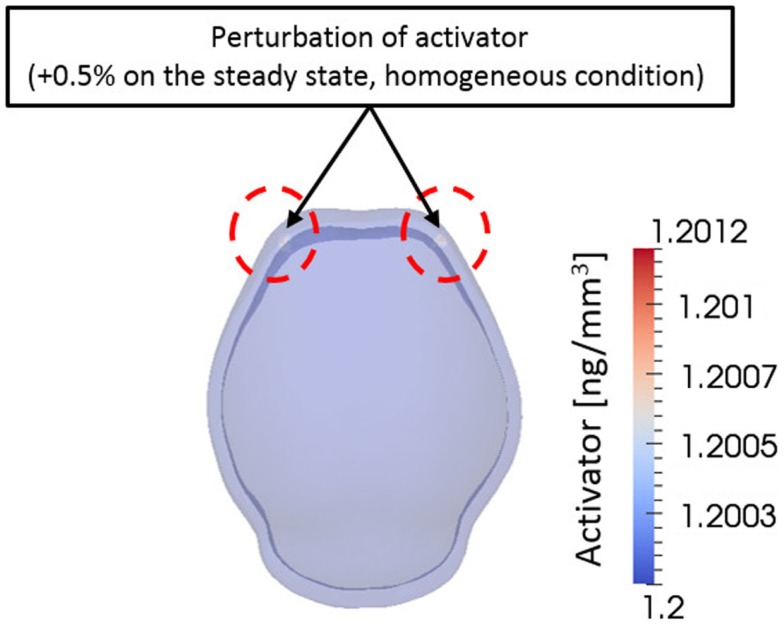
**Initial condition**. A small perturbation of activator on two points on the frontal side of domain was given as an initial condition. Elsewhere the domain has homogeneous concentration of activator and inhibitor. This condition is based on the biological observation showing that mesenchymal condensations initiate at the supra orbital region just above the globe of the eye and develop into right and left frontal bones.

From a computational point of view, initial conditions are a significant aspect to consider in a simulation. The model in this study also depends on initial concentration of activator and inhibitor molecules. Different initial concentrations of activator and inhibitor result in significant changes in the final bone growth patterns. The effect of various initial conditions can be seen in Supplementary Material. Experimental research reveals that mesenchymal condensations that will form the primary centers of ossification initiate at the supra orbital region just above the globe of the eye and develop into right and left frontal bones (Tubbs et al., 2012). Based on these observations, we initiated a small perturbation of activator (0.5% of steady state condition) only at two points corresponding to the location of the right and left frontal bones on the domain. Consequently, the entire surrounding domain has steady state concentration of activator and inhibitor [*C_a0_* = 1.20 ng/mm^3^, *C_h0_* = 1.44 ng/mm^3^ in equation (3a)] except the two points as shown in Figure 8. Zero-gradient of concentration of molecules was applied as boundary condition at domain boundaries, which means there is no flux of molecules by diffusion across the boundaries.

## 3 Results

### 3.1 Primary ossification centers

The reaction–diffusion model [equations (1a) and (1b)] and the cell differentiation model [equations (5a) and (5b)] were solved using the finite volume method and changes in concentrations of activator, inhibitor, and osteoblasts in the domain were examined over time. We assume mesenchymal cells are present near E0. This assumption is not the case in real development because there is neither brain formed nor mesenchymal cells surrounding the brain, but helps to establish a modeling process. Simulation using a brain shape at earlier time is planned in a subsequent research.

**Figure 9:**
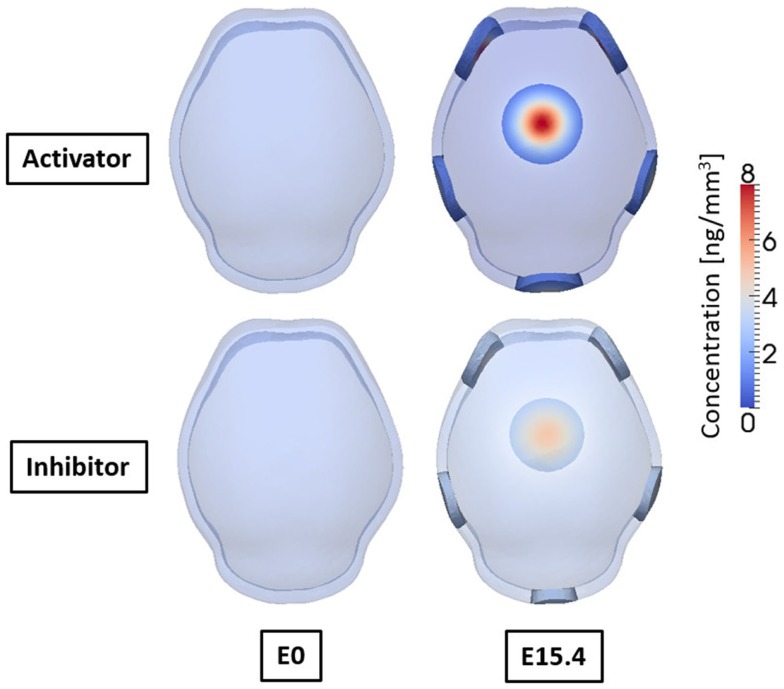
**Concentrations of activator and inhibitor at embryonic day 0 and 15.4 from simulation result**. Small perturbation of activator at initial time increases through regulatory reaction between activator and inhibitor, and finally makes a specific pattern of concentration of activator and inhibitor after 15.4 days. Activator and inhibitor are in phase. Six regions of high concentration of the molecules appear, two on the front, two on the side, one on the rear, and one on the top of the domain.

**Figure 10:**
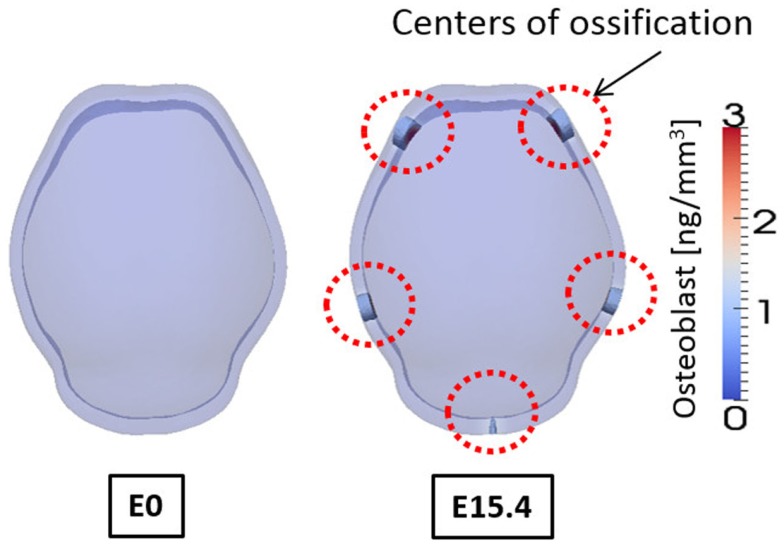
**Concentration of osteoblast at embryonic day 0 and 15.4 from simulation result**. Osteoblasts are differentiated in the regions where concentration of activator is high. And these regions of osteoblasts can develop into mineralized bone that is primary centers of ossification. Osteoblasts are not differentiated on the top of the domain although concentration of activator is high there, due to the spatial effect on cell differentiation in our model. Five primary centers of ossification and this agree well with experimental observation showing two frontal, two parietal, and a single interparietal bones.

Figure 9 shows the concentrations of activator and inhibitor at time 0 and after E15.4. At time E0 activator and inhibitor are distributed uniformly through the domain with only 0.5% of disturbances of activator on the two points at frontal side. These small perturbations at time E0 increase over time by regulatory reaction between activator and inhibitor to make a specific pattern of concentration in the domain so that six highly concentrated regions appear as shown in result at E15.4. Concentrations of activator and inhibitor are in phase and reveal the key feature of activator–inhibitor models: the activator is enhanced by itself and activates the inhibitor, but the inhibitor acts only to inhibit the activator. Based on our hypothesis that differentiation of mesenchymal cells to osteoblasts will occur local to points of elevated concentration of activator, our cell differentiation model [equations (5a) and (5b)] predicts five primary centers of ossification as shown in Figure 10.

Figure 10 shows the concentrations of osteoblasts at E0 and E15.4. We consider the regions where the concentration of osteoblast exceeds a threshold value (1.0 ng/mm^3^) to develop into a bony element via the formation of primary ossification centers and these regions are shown as opaque while other regions are translucent in Figure 10. Compared to the distribution of the activator (Figure 9), the primary ossification centers appear where the concentration of the activator is high except the top of the domain. The top of the domain has high concentration of the activator but due to spatial effect on cell differentiation, which we assume in the model [governed by equations (5a) and (5b)] ossification does not initiate in this region. Biologically, this may be an important observation as this is the location where the anterior fontanelle (soft spot) forms in developing heads. Five primary ossification centers form and are located on the right and left side of the anterior region, on the right and left side of the middle region, and in the center of the rear side of the domain, respectively. These computationally generated centers of ossification correspond with the biological location of the right and left frontal bones, right and left parietal bones, and interparietal bone, respectively.

### 3.2 Bone growth

**Figure 11:**
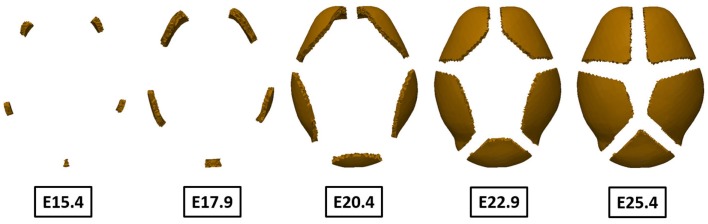
**Change of region of high concentration of osteoblast over time**. The regions originally marked by the differentiation of osteoblasts expand from the primary centers of ossification over time. Because these osteoblasts differentiate into osteocytes and eventually become trapped within mineralized bone, it can be said to show pattern of bone growth. The results agree well with experimental observation showing two frontal bones, two parietal bones, and one interparietal bone. Sutures form between bones as bones grow according to repulsive effect between bones in our model.

The bone growth model [equations (6) and (7)] was solved using the results of primary ossification centers as an initial condition and the change of concentration of differentiated osteoblast was examined. Figure 11 shows only regions where concentration of osteoblasts exceeds the threshold value (1.0 ng/mm^3^), which can develop into bone eventually. It shows the regions of high concentration of osteoblasts expand from the primary ossification centers from E15.4 to E22.9. The region of condensed osteoblasts on the posterior end of the domain grows slow compared to the other four regions because this primary ossification center is the last to form and is smaller in size. Spaces between the ossifying regions become reduced as the bones grow but they do not meet each other because of the repulsive effect that occurs between bones in the model. This is an important aspect of our model, which may model suture formation.

## 4 Discussion

The number and locations of ossification centers found in the simulation results (Figure 10) agree well with experimental data showing five ossification centers representing the two frontal, the two parietal, and the single interparietal bones of the cranial vault (Figures 2 and 7B). Based on the fact that these results were computed by not specifying any locations, which would eventually become ossification centers, but by only solving the diffusion–reaction model with proper initial perturbations and model parameters, the computational framework using Turing’s model can be seen as one reasonable approach for exploring processes required for the formation of primary ossification centers and for cranial vault growth.

The results of simulation of bone growth (Figure 11) show that the region of osteoblasts expands from the ossification centers. Our bone growth model explains this process as differentiation of adjacent undifferentiated mesenchymal cells near primary centers of ossification into osteoblasts by the action of a morphogen that is expressed from pre-existing osteoblasts and diffused in space with time. This is in agreement with previous research that demonstrates that the cells that add to the primary ossification centers by differentiating into osteoblasts come from the condensations rather than being recruited from other mesenchymal populations surrounding the brain (Yoshida et al., 2008; Ting et al., 2009). Once differentiated, osteoblasts function to produce osteoid along collagen bundles and then mineralize that matrix eventually becoming trapped within mineralized lacunae and differentiating into osteocytes. Here, we have not modeled the entrapment of osteocytes into a mineralized matrix, but the expansion of regions of high concentration of osteoblasts is considered as bone growth. Results shown at E20.4 (Figure 11) are representative of data at P0 because gestation period of mouse, although variable, is approximately 20 days. The simulation result shows smaller volume of bones compared to experimental data (Figure 2), which means a slower growth rate. In addition, the relative size and shapes of the individual bones are somewhat different from experimental observations. These differences can be overcome by subtle adjustment of model parameters and, importantly, the adjustments may add to our knowledge of the processes involved in the formation of ossification centers and their growth. The effects of different model parameters on the simulation are presented in Supplementary Material. Our hypothesis that morphogens from one bone element inhibits the growth of contiguous bones [equation (6)], may not be true, but can be tested experimentally. That our results show suture formation similar to what is observed experimentally suggests that suture formation involves some kind of mechanism of repulsive factors between bones, and this does not counter a hypothesis of boundary formation between cellular compartments that serve as signaling interfaces as suggested by others (Merrill et al., 2006; Ting et al., 2009). Here, we suggest that the factors driving the formation of sutures may include chemical substances or mechanical stimuli by the growing brain or by opposing bones as the gaps between them narrow. Since the processes that control normal suture development and the mechanisms underlying abnormal premature suture closure are not well understood, further research into the effects of mechanical stress on bone growth is warranted.

Although the simulation captures many features of the developmental process, it has some limitations as well. Key molecules play important roles in cell differentiation and their identity, as well as the real values of these parameters remain to be determined. We expect this to be achieved in a follow-up study that will elucidate the cellular-level changes that occur in cranial development providing the basis for joining molecular cues and cell behavior with 3D shape changes that occur during ontogeny. The number of cells in initial ossification centers, rate of OLC differentiation and proliferation, intracranial pressure gradients from growth induced skull-soft tissue interaction, and rate of suture closure can be parameterized and modified in the model. The results can be continually quantitatively compared to our extensive image archive of developing cranial soft and hard tissues.

In this study, the computational domain is fixed in both size and shape while in biological systems the surface on which bone development occurs expands and changes shape as the underlying brain grows. Indeed, the size and shape of the domain does affect the number and location of ossification centers because diffusion is strongly related with geometry of the domain. In the future, we plan to let the shape of the computational domain change (or “grow”) over time. This would offer a more realistic model, but we realize that our diffusional parameters will need to change over time as well. Some parts of our model are based on the experimental observation rather than clear mechanical mechanisms of how sutures form, why ossification does not occur at the top of the domain and how subtle, complicated shape of each bone forms. We expect that mechanical stimuli such as pressure and stress may affect these phenomena and they will be a focus of a future study. In a forthcoming study, our proposed framework will be improved by including effects of brain growth and mechanical stimuli (e.g., stress) between the brain and growing bone and pressure distribution in head.

## 5 Conclusion

Growth of the cranial vault is coordinated through tissue–tissue interactions between the brain, the developing meninges, the bone primordia, and the cranial sutures (Han et al., 2007), but the specifics are not well defined. Here, the processes associated with formation of primary centers of ossification and bone growth were mathematically modeled and solved using the finite volume method. The results show that five primary ossification centers form at positions like those identified in experimental data (Figure 10). Our results reveal bone growing from the primary ossification centers forming sutures between bones (Figure 11). Our study shows that the development of the cranial vault can be numerically simulated using the established computational framework. We expect that changes in model parameters, when examined in parallel with laboratory experimentation, will help clarify some of the key players and mechanisms of skull development, both normal and pathological.

## Conflict of Interest Statement

The authors declare that the research was conducted in the absence of any commercial or financial relationships that could be construed as a potential conflict of interest.

## Supplementary Material

The Supplementary Material for this article can be found online at http://www.frontiersin.org/Journal/10.3389/fbioe.2015.00024/abstract
