# Supplementary material for: A Computational Analysis of Bone Formation in the Cranial Vault in the Mouse
Source: Front Bioeng Biotechnol. 2015 Mar 19;3:24. doi: 10.3389/fbioe.2015.00024 (PMC4365500; doi:10.3389/fbioe.2015.00024)
Supplement: Supplementary file 1 [file Data_Sheet_1.PDF]

A Computational Analysis of Bone Formation in the Cranial Vault in the Mouse

Chanyoung Lee [1], **Joan T. Richtsmeier** [2] **and Reuben H. Kraft** [1,*]

[1] *The Penn State Computational Biomechanics Group, Department of Mechanical and Nuclear Engineering, Pennsylvania State University, University Park, PA, USA*
[2] *Department of Anthropology, Pennsylvania State University, University Park, PA, USA*

Correspondence*:
Reuben H. Kraft
The Penn State Computational Biomechanics Group, Department of Mechanical and Nuclear Engineering, Pennsylvania State University, 320 Leonhard Building, University Park, PA, 16802, USA, reuben.kraft@psu.edu

APPENDIX A: EFFECTS OF MODEL PARAMETERS ON THE FORMATION OF OSSIFICATION CENTERS

The reaction-diffusion model (Eq. (1a) and (1b)) and cell differentiation model (Eq. (5a) and (5b)) were solved with three different sets of model parameters to examine the effects of the parameters on the formation of primary ossification centers. Three parameter sets have different diffusion rate of activator and inhibitor while other parameters are same. Diffusion rates of the molecules are relatively slow in Case P-1, medium in Case P-2 and fast in Case P-3 (Table S 1). Case P-2 (medium diffusion rate) is the parameter set which is used in main section of this study.

Table S1. Three different sets of diffusion rate

Parameters		Value			Units
		Case P-1	Case P-2	Case P-3	
Activator Diffusion	D_a	1.8×10^{-6}	2.5×10^{-6}	3.2×10^{-6}	mm^2/s
Inhibitor Diffusion	D_h	1.8×10^{-4}	2.5×10^{-4}	3.2×10^{-4}	mm^2/s

With the same initial condition which is used in the main section, three cases of parameters lead different numbers and locations of primary centers of ossification as shown in Fig. S1. Figure S1 presents the concentrations of activator and osteoblast at E15.4 in three different cases. Because the diffusion of the molecules are slow in Case P-1, the molecules cannot go far from front side of the domain on which perturbations start so high concentrated regions of activator forms forward (Fig. S1-(a)) compared to Case P-2 (Fig. S1-(b)) which has medium diffusion rate. Therefore primary ossification centers also form forward in Case P-1 compared to Case P-2. Faster diffusion speed of molecules (Fig. S1-(c)) result in high concentrated region of activator and osteoblast on the further rear. It also affects to the number of primary centers of ossification as one of ossification centers which are expected based on experimental data does not appear. These results show it is significant to determine proper model parameters to study bone growth when using the computational model. On the other hand the results also show a possibility to study defect mechanism by using the model with abnormal model parameters.

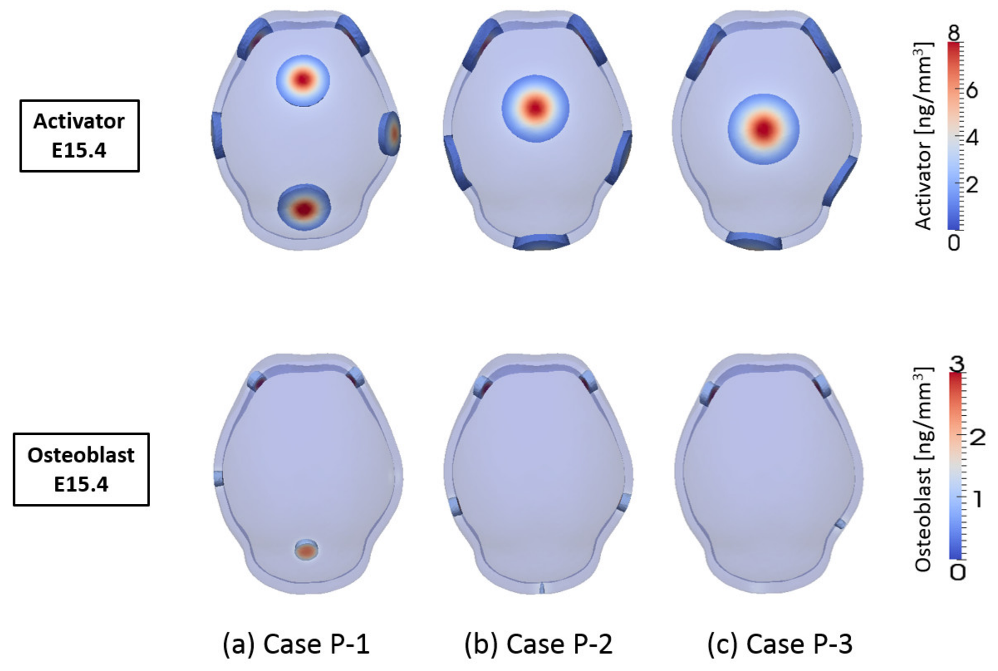

Figure S 1. Simulation results of concentration of activator and osteoblast at E15.4 with three different sets of model parameters. In the case of slow diffusion rate ((a) Case P-1) high concentrated regions of activator forms forward therefore primary ossification centers also form forward compared to in (b) Case P-2. Faster diffusion speed of molecules ((c) Case P-3) result in high concentrated region of activator and osteoblast on the further rear.

APPENDIX B: EFFECTS OF INITIAL CONDITION ON THE FORMATION OF OSSIFICATION CENTERS

The reaction-diffusion model (Eq. (1a) and (1b)) and cell differentiation model (Eq. (5a) and (5b)) were solved with three different sets of initial condition to examine the effects of the initial perturbations of molecules to the formation of primary ossification centers.

Figure S2 shows the three cases of initial condition and following results. Case I-1 (Fig. S2-(a)) is the initial condition used in main section of this study, which has perturbations of activator (0.5% of steady state condition) only on the two points at frontal side of domain. Case I-2 and Case I-3 show randomly distributed activator with 0.5% of perturbations but detailed distributions are different from each other. The same model parameters were used as used in main section of this paper. Figure S2 presents the concentration of activator and concentration of osteoblast at E15.4 with the three different initial condition. Results show that different initial distributions of activator lead different numbers and locations of primary ossification centers. In Case I-1 where initial perturbations of activator are on the symmetrical position, the high concentrated regions of activator and primary ossification centers form symmetrically (Fig. S2-(a)). However in Case I-2 and Case I-3, initially asymmetric distributions of activator result in asymmetric distribution of high concentrated regions and primary ossification centers (Fig. S2-(b) and (c)).

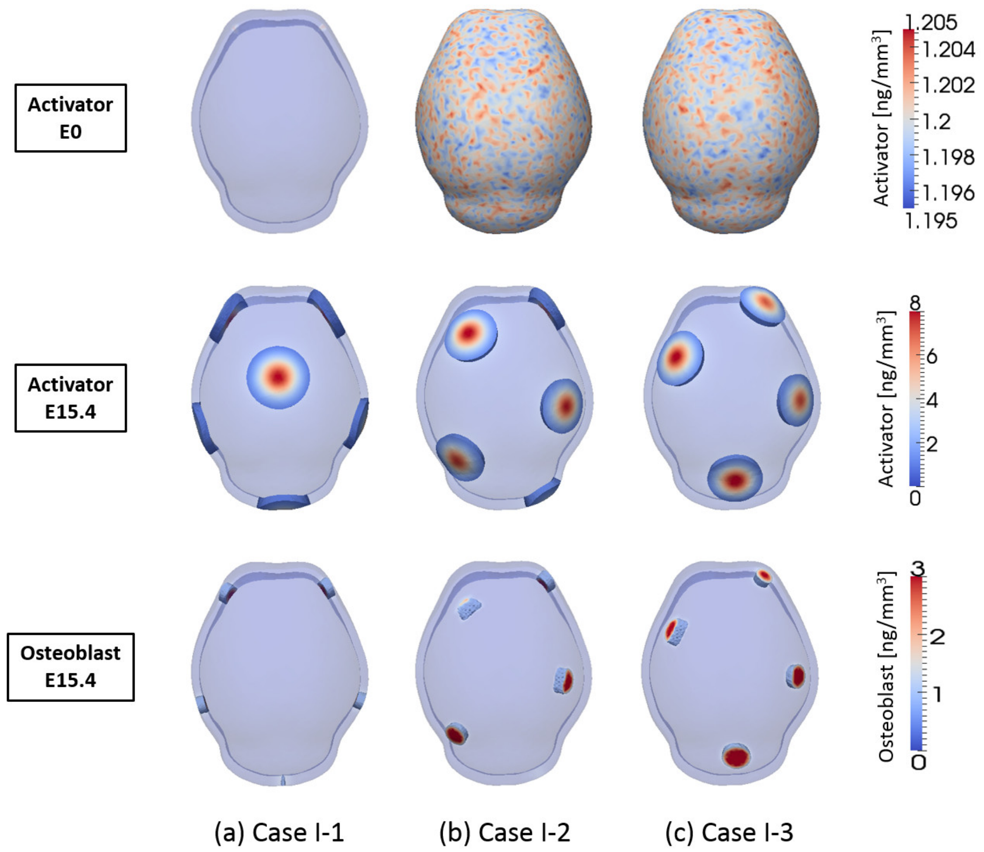

Figure S 2. Distribution of activator and osteoblast with different initial condition. Different initial distributions of activator lead different numbers and locations of primary ossification centers. Initial condition of (a) Case I-1 has perturbations of activator only on the two points at frontal side of the domain. As initial perturbations are on the symmetrical position, the distributions of activator and osteoblast are symmetrical at E15.4. (b) Case I-2 and (c) Case I-3 have randomly distributed activator as initial conditions. Initially asymmetric distributions of activator result in asymmetric distribution of activator and osteoblast at E15.4.
